# Working memory and arithmetic impairments in children with FMR1 premutation and gray zone alleles

**DOI:** 10.1590/1980-5764-DN-2021-0035

**Published:** 2022

**Authors:** Aline Aparecida Silva Martins, Giulia Moreira Paiva, Carolina Guimarães Ramos Matosinho, Elisângela Monteiro Coser, Pablo Augusto de Souza Fonseca, Vitor Geraldi Haase, Maria Raquel Santos Carvalho

**Affiliations:** 1 Universidade Federal de Minas Gerais, Intituto de Ciências Biológicas, Departamento de Genética, Ecologia e Evolução, Postgraduate Program em Genética, Belo Horizonte MG, Brazil.; 2 Universidade Federal de Minas Gerais, Intituto de Ciências Biológicas, Departamento de Genética, Ecologia e Evolução, Belo Horizonte MG, Brazil.; 3 Universidade Federal de Minas Gerais, Faculdade de Filosofia e Ciências Humanas, Departamento de Psicologia, Belo Horizonte MG, Brazil.; 4 Universidade Federal de Minas Gerais, Instituto de Ciências Biológicas, Programa de Pós-Graduação em Neurociências, Belo Horizonte MG, Brazil.; 5 Fundação Oswaldo Cruz, Instituto René Rachou, Departamento de Informática de Biossistemas e Genômica, Belo Horizonte MG, Brazil.; 6 Universidade Federal de Minas Gerais, Faculdade de Medicina, Postgraduate Program em Saúde da Criança e do Adolescente Belo Horizonte MG, Brazil.; 7 Universidade Federal de Minas Gerais, Faculdade de Filosofia e Ciências Humanas, Departamento de Psicologia, Postgraduate Program em Psicologia, Belo Horizonte MG, Brazil.; 8 Instituto Nacional de Ciência e Tecnologia em Cognição, Comportamento e Ensino, São Carlos SP, Brazil.

**Keywords:** Learning, Dyscalculia, Working Memory, FMR1, Aprendizagem, Discalculia, Memória de Trabalho, FMR1

## Abstract

Expansive mutations in familial mental retardation 1 (*FMR1*) gene have been associated with different phenotypes. Full mutations are associated with intellectual disability and autism spectrum disorder; premutations are associated with math learning difficulties and working memory impairments. In gray zone, neuropsychological development has not yet been described.

**Objectives:**

This study aimed to describe the frequency of *FMR1* premutation and gray zone alleles in a school population sample representing a broad spectrum of variation in math achievement and detail school achievement and cognitive performance in the children identified with *FMR1* premutation or gray zone alleles.

**Methods:**

We described a two-phase study. In the first phase, 2,195 school-age children were screened for math achievement. In the second phase, 378 children with normal intelligence were neuropsychologically assessed and genotyped for *FMR1*. Of these, 121 children (61 girls) performed below percentile 25 in mathematics (MD group) and 257 children (146 girls) performed above percentile 25 (control group).

**Results:**

Four pupils presented expanded alleles, one premutation and three gray zone alleles. The girl with the premutation and one boy with a gray zone allele presented impairments in working memory and arithmetic performance below percentile 6, compatible with the diagnosis of developmental dyscalculia. These children’s difficulties were not associated with inaccuracy of nonsymbolic number representations or literacy impairments. Dyscalculia in these children seems to be associated mainly with working memory impairments.

**Conclusions:**

*FMR1* expansions in the gray zone may contribute to dyscalculia in otherwise healthy and normally intelligent children.

## RESUMO.

Mutações expansivas no gene *FMR1* têm sido associadas a diferentes fenótipos. Mutações completas estão associadas a deficiência intelectual e transtorno do espectro do autismo; pré-mutações, com dificuldades de aprendizagem de matemática e comprometimentos de memória de trabalho. Na zona cinzenta o desenvolvimento neuropsicológico ainda não foi descrito.

**Objetivos:**

Descrever a frequência de alelos pré-mutados e zona cinzenta em uma amostra escolar que representa amplo espectro de variação do desempenho em Matemática e detalhar o desempenho escolar e cognitivo em crianças identificadas com alelos pré-mutados ou zona cinzenta.

**Métodos:**

Aqui, descrevemos um estudo de duas fases. Na primeira fase, 2.195 crianças em idade escolar foram selecionadas para desempenho em Matemática. Na segunda fase, 378 crianças com inteligência normal foram avaliadas neuropsicologicamente e, em seguida, por genotipagem *FMR1*.

**Resultados:**

Tiveram desempenho abaixo do percentil 25 em Matemática (grupo DM) 121 crianças (61 meninas), e tiveram desempenho acima do percentil 25 (grupo controle) 257 crianças (146 meninas). Quatro alunos apresentaram alelos expandidos, sendo uma pré-mutação e três alelos da zona cinza. A menina com a pré-mutação e um menino com o alelo da zona cinza apresentaram prejuízos na memória de trabalho e desempenho aritmético abaixo do percentil 6, compatíveis com o diagnóstico de discalculia do desenvolvimento. As dificuldades dessas crianças não foram associadas à imprecisão de representações não simbólicas de números ou deficiências de alfabetização. A discalculia nessas crianças parece estar associada principalmente a deficiências da memória de trabalho.

**Conclusões:**

Em conclusão, expansões na zona cinzenta do *FMR1* podem contribuir para a discalculia em crianças saudáveis com inteligência normal.

## INTRODUCTION

Difficulties with math learning are associated with poor psychosocial outcomes, such as lower wages, unemployment, and externalizing and internalizing psychopathology^1^. Problems with math learning are characterized as dyscalculia, when the performance in standardized tests is below the percentile rank 5 (PR5), and as Math difficulties (MD) when the performance is below PR25^2^. Prevalence rates correspond to the cutoff scores used^3^. Evidence for a genetic component in dyscalculia and MD stems from familial aggregation and twin studies indicating heritability in the 40–60% range, as well as from associated molecular-genetic markers^4^. In addition, dyscalculia is part of the phenotype of some genetic conditions, including typical and atypical 22q11.2DS, Williams-Beuren and Turner syndromes, and neurofibromatosis type 1^5^.

Dyscalculia was also described in clinical samples of persons having a premutation in the familial mental retardation 1 (*FMR1*) gene^6^. Normally, this gene contains a CGG repetitive element on its 5′-untranslated region (5’-UTR). This CGG repeat is polymorphic, and according to the number of CGGs, the *FMR1* gene presents four allelic classes: normal (6–44 CGG), gray zone (45–54 CGG), premutation (55–199 CGG), and full mutation alleles (>200 CGG)^7^. In some studies, the range between 41 and 54 CGG repeats was considered an expanded gray zone^8^.

*FMR1* mutations have been associated with several phenotypes. *FMR1* full mutations cause the fragile X syndrome (FXS), which is characterized by intellectual disability and working memory (WM) deficits in boys, and borderline to normal IQ and autism associated with obesity from the second decade of life on in girls^9^. *FMR1* premutations have been associated with FXTAS, premature ovarian failure (POF), and Parkinson’s disease^10^. Individuals with premutation may have deficits in cognitive functions such as processing speed, WM, executive functions, phonological, visuospatial processing, numerical magnitude comparison, long-term memory, and learning deficits^11-16^. Boys with premutation present higher rates of attention deficit/hyperactivity disorder, autism spectrum disorder, and intellectual disability^17^. Girls with a premutation allele may also have difficulties in basic numeric processing and arithmetic such as enumeration, numerical transcoding, and calculation, similar to those observed in dyscalculia^6,18,19^. Additionally, adults may have psychiatric problems, such as anxiety and depression^20^. Individuals with gray zone alleles are usually asymptomatic, but may present impairments in short-term memory and WM. In addition, dyslexia has been reported in two males with gray zone alleles in a family study^21^. However, to the best of our knowledge, there are no data in the literature associating gray zone alleles with developmental dyscalculia or MD.

Typically, studies reporting learning difficulties in children with premutations and gray zone alleles are based on clinical samples. Such studies tend to capture most prominent phenotypes and may not represent the spectrum of phenotypic variations present in the general population. School samples are less prone to such biases. However, no study has investigated the contribution of *FMR1* premutation or gray zone alleles to dyscalculia in population samples of school-age children using a detailed neuropsychological assessment. Here, we described the frequency of *FMR1* premutation and gray zone alleles in a school population sample representing a broad spectrum of variation in math achievement. The school achievement and cognitive performance was further detailed in the children identified with *FMR1* premutation or gray zone alleles.

## METHODS

### Bioethical approach

The study complied with the Helsinki guidelines for research with human participants and was previously approved by the ethics board of the Universidade Federal de Minas Gerais (COEP-UFMG). Informed consent was obtained in written form from parents/guardians and orally from children.

### Participants

Data were obtained from a demographically based sample of children from 6 to 14 years attending public schools in Belo Horizonte, Brazil. Initially, 2,195 children participated in a screening phase, responding to group-administered tests of intelligence and school achievement (Table 1). Children with intelligence above PR15 were invited to participate in a second phase of individual neuropsychological assessment, using the following exclusion criteria: gemelarity, chronic diseases (e.g., diabetes, sickle cell anemia, and epilepsy), autism spectrum disorder, monogenic or chromosomal genetic syndromes, and maternal alcohol/drug use during pregnancy. A total of 378 pupils participated in the second phase of the study and were genotyped for the *FMR1* CGG repeat.

**Table 1:** Sociodemographic characteristics of participants.

		Group assessment			Individual assessment		
		Total	Control	MD	Total	Control	MD
n		2,195	1,761	434	378	257	121
Female sex, n (%)		1,195 (54.4)	987 (56.05%)	208 (47.92)	207 (54.7)	146 (56.8)	61 (50.4)
Age (years)	Mean	9.08	9.04	9.26	9.36	9.35	9.40
	SD	1.45	1.40	1.62	1.26	1.17	1.45
Grade	Mean	3.89	3.85	4.05	4.16	4.14	4.19
	SD	1.21	1.21	1.23	1.07	1.05	1.12

MD: Math difficulties.

Performance in the Arithmetic subscale of the Brazilian School Achievement Test (TDE)^22^ allowed to classify participants into a group of broadly defined MD with performance below PR25 and a control group with performance at above PR25. In the second phase, stricter diagnostic criteria (PR<6–7) informed by the individual neuropsychological assessment were applied to classify math achievement.

A total of 378 children completed both phases of the neuropsychological evaluation, 121 children (61 girls) were identified as having MD. To the control group were assigned 257 children (146 girls).

### Instruments

Neuropsychological testing was conducted at the schools by specially trained psychology research assistants. Tests were applied in groups in the first phase and individually in the second phase. The neuropsychological protocol is shown in Table 2. A few additional tasks were applied in individuals with *FMR1* expanded alleles.

**Table 2:** Instruments used in the neuropsychological assessment.

Study phase	Construct	Instrument	Description
Group assessment	Intelligence	Raven’s Coloured Progressive Matrices (CPM)^23^	Nonverbal reasoning abilities
	School achievement	TDE — Arithmetic and word Spelling subtests^22^	Standardized test of school achievement, including subscales for mathematics (arithmetic operations) and word spelling
Individual assessment	Verbal and nonverbal short-term and working memory	WISC-III Digits^24^	Forward testing: representational ability Backward testing: working memory Span of apprehension: working memory Total score: attentional processes
		Corsi Blocks^25^	
	Phonological processing	Phoneme elision task^26^	Blending of a new word when a specific phoneme is deleted: cRop → cop, Brisk → risk, Cup → u
	Numerical and arithmetic abilities	Nonsymbolic magnitude comparison task^27^	Internal Weber fraction (w) indexing accuracy of nonsymbolic number representation
		Arabic number reading^28^	Numbers up to 4 digits with increasing degrees of transcoding difficulty
		Arabic number dictation^28^	
		Simple single-digit addition^27^	Simple additions: operations with result below 10 (i.e., 3+5)
		Simple single-digit subtraction^27^	Simple subtraction: operands were below 10 (i.e., 9−6)
		Simple single-digit multiplication^27^	Simple multiplications: results below 25 and/or number 5 as one of the operands (i.e., 2×7, 5×6)
		Simple additive arithmetic word problems^27^	Story problems with single-digit operands and results ranging from 2 to 9 (e.g., “Annelise has 9 cents. She gave 3 to Pedro. How many cents does Annelise have now?”)

TDE:*Teste de Desempenho Escolar* (School Achievement Test); WISC-III: Wechsler Intelligence Scale for Children III.

#### FMR1 genotyping

Genomic DNA was extracted from peripheral blood or saliva samples, using a salting out protocol^29^. Genotyping of the *FMR1* CGG repeats was performed using the AmplideX FMR1 PCR Reagents RUO Kit (Asuragen, EUA). This method allows accurate identification of expanded *FMR1* alleles^30,31^. Polymerase chain reaction (PCR) products were separated using an ABI 3730 DNA Analyser (Thermo Fisher Scientific, USA). Profiles were evaluated using the GeneMarker version 2.6.2 software (SoftGenetics, USA)*. FMR1* genotyping was repeated to confirm results, when gray zone, premutation, or full mutation alleles were detected.

### Statistical analyses

Frequencies of gray zone and premutation alleles in the MD and control groups were compared using the Fisher’s exact test. Neuropsychological results of each individual with expanded *FMR1* alleles were compared to those of subgroups of controls, referred to as comparison groups, controlling for sex, age, school grade, and socioeconomic status. The z metric was chosen for comparisons, with a cutoff of normality at z=-1.5 (PR6–PR7)^32^.

## RESULTS

### *FMR1* genotypes

The results of the *FMR1* genotyping for the 378 participants on the individual neuropsychological assessment are shown in Table 3.

**Table 3:** Alleles identified in Math difficulties and control groups (n=378).

Sex	Alleles/group	Normal n (number of CGGs)	Expanded gray zone n (number of CGGs)	Gray zone n(number of CGGs)	Premutation n (number of CGGs)	Full mutation n (number of CGGs)
Girls	MD (n=61)	60 (19–37 CGGs)	–	–	1 (57 CGGs)	–
	Controls (n=146)	141 (10–40 CGGs)	4 (41–43 CGGs)	1 (47 CGGs)	–	–
Boys	MD (n=60)	59 (18–36 CGGs)	–	1 (46 CGGs)	–	–
	Controls (N=111)	110 (16–38 CGG)	–	1 (45 CGGs)	–	–

MD: math difficulties.

One premutation allele was detected in a girl from the MD group (1:61 considering only girls and 1:121 considering boys and girls). Three children presented alleles in the gray zone: one boy and one girl in the control group (1:111 and 1:146, respectively) and one boy in the MD group (1:60 considering boys and 1:121 considering boys and girls). In addition, four children in the control group (4:146) presented alleles with 41–44 CGG, which belong to the expanded gray zone range (Table 3). These four children have normal results in the neuropsychological assessment and will not be further discussed. The most frequent allele had 30 CGG repeats, except for the boys in the MD group for whom the 31 CGG repeat allele was the most frequent (Supplementary Figures 1–4). As expected, considering the inclusion criteria of intelligence above PR15, no *FMR1* full mutations were observed.

### Neuropsychological performance of children with expanded FMR1 alleles

**Figure 1:**
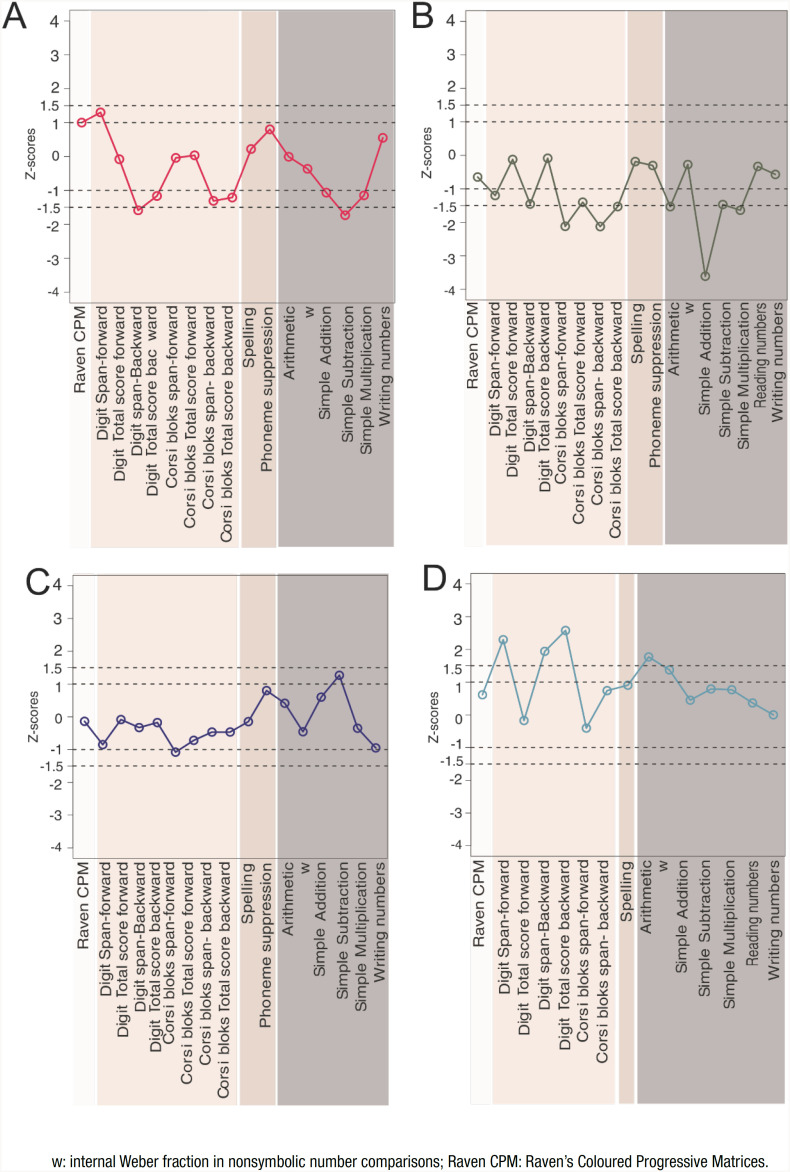
Neuropsychological performance of the children with FMR1 premutation and gray zone alleles. (A) Child 1 (premutation); (B) Child 2; (C) Child 3; (D) Child 4. Deficits in individual tests were assumed when scores < or = -1.5 SD (PR6–PR7).

Quantitative results of the neuropsychological performance of each child with a premutation or a gray zone expanded *FMR1* allele are presented in Figure 1. Table 4 presents descriptions of the neuropsychological phenotypes for each child.

**Table 4:** Genotype and phenotype characteristics of children with expanded *FMR1* alleles.

Child	CGG repeat	Genotype classification	Math achievement	Neuropsychological phenotype description
1	57	Premutation	MD	Female, 9 years 4 months, fourth grade. Normal intelligence (70th percentile) and word spelling. Slow and effortful single-digit calculation using ineffective counting strategies. Unable to execute single-digit multiplications and divisions. Borderline accuracy (w) of nonsymbolic numerical representations. Deficits in backward Digit Span and backward Corsi blocks (Figure 1A).
2	46	Gray zone	MD	Male, 10 years 1 month, fourth grade. Typical developmental and medical antecedent. Intelligence in the 30th percentile and normal word spelling. Difficulties in Arabic number dictation with mostly syntactic errors related to place value understanding. Slow and effortful Arabic single-digit calculations, requiring counting strategies. No understanding of multiplication operations. Severe difficulties in simple, single-digit narrative arithmetic problems. Tendency to perseverate on the problem statement. Difficulties with clock reading. Normal nonsymbolic numerical representation accuracy (w)l. Difficulties with both Digit and Corsi blocks tests of WM (Figure 1B). Reexamination at age 16 years 3 month: he complains of low interest in social interactions, school disinterest, difficulty in all subjects, but more intense in mathematics. Persistence of MD observed in the first examination with lack of multiplication facts memorization and use of counting strategies to solve single-digit arithmetic problems.
3	45	Gray zone	Control	Male, 9 years 3 months, fourth grade. Normal average intelligence word spelling. No impairments in single-digit calculation. Still acquiring the multiplication tables. No other neuropsychological impairments (Figure 1C).
4	47	Gray zone	Control	Female, 12 years 11 months, sixth grade. Normal developmental and medical antecedents. Normal neuropsychological examination (Figure 1D).

## DISCUSSION

The investigated hypothesis was that dyscalculia is a phenotype associated with expanded *FMR1* alleles in demographically recruited school-age children. In a sample of 378 children with normal intelligence and no chronic disease and/or neurodevelopmental disorder, 4 children were identified as having expanded *FMR1* alleles. Here, we compared the observed frequencies of the premutation and gray zone alleles with those described in the literature and describe the neuropsychological profiles of these children.

### Frequency of *FMR1* premutation or gray zone alleles in the sample

Our first question was whether the frequency of premutation or gray zone alleles was similar to that described in the literature. In samples of persons with intellectual disability, the contribution of *FMR1* full mutations has been estimated to be 1–6%^33,34^. The frequency of *FMR1* full mutations was estimated to be approximately 1:5000 in a screening sample composed of over 36,000 newborn males^35^. Therefore, having an *FMR1* full mutation entails a risk for intellectual disability that is 50–300 times higher than the risk in the general population. Considering the exclusion criteria implemented here and the sample size, we did not expect to identify any *FMR1* full mutations in the current sample.

Our focus was on the contribution of *FMR1* premutation or gray zone alleles for learning difficulties and related phenotypes. In the literature, the frequency of premutation and gray zone alleles was investigated in a sample composed of approximately 20,000 male and female adults representing the general population^36^. According to this study, the frequency of premutation in females was 1:148 and that in males was 1:290; the frequency of gray zone alleles (45–54 repeats) was 1:33 females and 1:62 males; and the frequency of “expanded” gray zone alleles (here defined as 41–54 CGG repeats) was 1:14 females and 1:22 males. In the current sample, the frequency of premutation, gray zone, and expanded gray zone alleles did not statistically differ from these values (results not shown).

### Neuropsychological impairments in demographically sampled cases of *FMR1* premutation and gray zone alleles

The second question was whether children sampled from the general population and identified with premutation, gray zone, or expanded gray zone alleles also have a neuropsychological profile resembling that described for some children from families in which FXS segregates, having similar alleles^19^. Two individuals were identified with performance below the PR6–PR7 in WM and arithmetic tasks. One child had a premutation and the another a gray zone allele. Both had normal intelligence and normal written language processing as assessed by word spelling, which excluded concomitant intellectual disability or a literacy acquisition disorder. In Child 1, phonological WM (Digit span backward) was below the cutoff score and spatial WM (Corsi blocks span backward) was in the lower normal range (1 SD below the average). The performance of Child 2 was at or below the cutoff in both phonological and spatial WM.

Both children also presented impairments in arithmetic abilities. Child 1 performed below the cutoff score in single-digit subtraction and more than 1 SD below the average in the other calculation tasks. Child 2 performed below the cutoff score for all single-digit operations. It is remarkable that these children presented difficulties with very basic arithmetic skills acquired in the first 3 years of schooling. It is also noteworthy that both the accuracy of nonsymbolic numerical representations (w) and number reading and writing were normal in these two children. This indicates impaired calculation together with spared number processing.

Math learning difficulties of varying degrees of severity have been reported in persons having premutations in families with FXS^19^. However, clinical samples tend to include more severe cases and to overestimate both the frequency and the impact of the difficulties. A possible solution to avoid such biases is to identify cases among the school population. Two studies have investigated the frequency of *FMR1* premutations and gray zone alleles in school-age children^37,38^. In the study by Murray and colleagues^37^, boys were identified in the schools by teachers or principals due to learning disability and no specific neuropsychological evaluation was conducted. In the study by Mazzocco and colleagues^38^, children who were identified in pediatric or developmental neuropsychology facilities as having normal intelligence and learning disabilities were screened for *FMR1* premutations and gray zone alleles. Neither of these screening studies specifically assessed WM and numerical/arithmetic abilities. Both studies may have included individuals with borderline intelligence or intellectual disability.

### Diagnosis of developmental dyscalculia

In both participants with expanded *FMR1* alleles (Child 1 and Child 2), performance in the single-digit calculation tasks was below the PR6–PR7. Both children also presented clinical evidence of impairments in very basic math-related school abilities. Qualitatively, both children had difficulty in understanding the concepts underlying the operations and used immature strategies, such as finger counting, to solve the most basic single-digit calculations. Thus, a diagnosis of developmental dyscalculia is justifiable according to the current nosological standard^3^.

### Cognitive mechanisms underlying math learning difficulties

Five main cognitive mechanisms have been proposed to explain difficulties in learning math in cases of developmental dyscalculia: (a) inaccurate nonsymbolic numerical magnitude representations in an approximate number system (ANS); (b) deficits in the access to nonsymbolic numerical representations from symbolic ones; (c) phonological processing deficits associated with developmental dyslexia; (d) visuospatial/visuoconstructional processing deficits associated with nonverbal learning disability; and (e) working memory/executive function impairments^5^. These cognitive mechanisms may be interpreted as endophenotypes in different combinations in children with dyscalculia, a heterogeneous condition, often occurring with developmental dyscalculia, attention deficit disorder, and other conditions^5^.

In this present study, neuropsychological results excluded impairments in written language and phonological processing in the two children with expanded *FMR1* alleles and dyscalculia. None of these two children presented difficulties with the accuracy of or access to nonsymbolic numerical representations. These results exclude a role for phonological and nonsymbolic numerical processing in their difficulties. Their difficulties were otherwise associated with impairments of both verbal and visuospatial WM.

The literature on females with FXS has disclosed an uneven cognitive profile of assets (verbal memory and analytic visual perception) and deficits (visuospatial and executive function)^39^. Other evidence indicates spared number reading/writing and rote counting abilities with deficits in magnitude judgments, mental number line judgments, understanding of counting principles, and basic addition in FXS girls^40,41^.

There are reports of individuals with *FMR1* premutation presenting deficits in cognitive functions such as WM, executive function, visuospatial perception, phonological processing, and reaction time^16,42,43^. Females with a premutation allele may present difficulties in basic numerical comprehension and numerical transcoding of mathematical questions and calculations^6,18,19^. It is remarkable, however, that the impairments described in premutation alleles are usually of lesser severity than required for the diagnosis of developmental dyscalculia.

Considering this literature, our neuropsychological results suggest that (a) WM impairments in individuals having *FMR1* expansions may play a role in difficulties learning math, as it has been previously reported; (b) ­accuracy of nonsymbolic numerical representations is normal in at least a subgroup of individuals with *FMR1* mutations; and (c) neuropsychological impairments are observed not only in FXS and premutation alleles but also, as described here, in individuals with gray zone alleles.

Our results must be cautiously interpreted. One limitation is the number of individuals having *FMR1* abnormal alleles detected. From the 2,195 children taking part in the initial population screening phase, 328 concluded the second phase of the study and, among these, only 4 had *FMR1* alleles in the abnormal range. These numbers seem small, reflecting the complexities underlying a population design necessary to detect *FMR1* expansions (due to their low frequency) and dyscalculia (due to the wide range of tests needed to diagnose it). However, it is important to highlight that the neuropsychological profile presented by these two children is typically seen in association with *FMR1* premutations, suggesting that the presence of developmental dyscalculia is not a fortuitous finding. The exclusion of low and borderline intelligence may have removed children with full mutations from the sample. However, the focus here was actually to ascertain children with normal intelligence and MD.

Child 1, a girl with a 57 CGG premutation, was the single individual identified with an *FMR1* premutation in our sample and she also presented developmental dyscalculia. According to the literature, these math difficulties should be expected only in individuals with more than 100 CGG repeats^11,18,44^. ­However, ­Lachiewicz and colleagues^6^ reported that girls with premutation alleles around 80–88 CGG repeats have lower arithmetic performance than women with alleles greater than 100 CGG repeats. The findings from ­Lachiewicz and coworkers and this study suggest that the typical neuropsychological profile may be present in the premutation range, independent of the number of CGG ­repeats.

Child 2, a boy with a 46 CGG gray zone allele was identified as having developmental dyscalculia. The other two children with gray zone alleles, as well as the four children with alleles in the 41–44 CGG range, had typical neuropsychological performance. Although other studies have investigated the contribution of *FMR1* full mutation and premutation alleles to MD, this is the first study to investigate their contribution in a sample of school-age children undergoing a detailed neuropsychological and cognitive-numerical assessment. We provided evidence that gray zone alleles may occur in individuals with normal neuropsychological profiles; whereas other individuals with gray zone or premutation alleles display specific neuropsychological deficits, characterized by low performance in WM and low math achievement, corresponding to a diagnosis of developmental dyscalculia. This suggests that other factors, in addition to the gray zone alleles, must be present to cause the developmental difficulties in learning math for these children.

The finding that two out of four children with an *FMR1* premutation and gray zone allele presented developmental dyscalculia associated with WM impairments suggests this is an important phenotype in *FMR1* expansions. In contrast, the frequency observed in this study, two children with abnormal *FMR1* alleles out of 121 children with MD, suggests that this gene makes an important contribution to MD and developmental dyscalculia. As impairments in WM were the most salient finding, it also suggests that WM impairment is an important endophenotype of developmental dyscalculia. Indeed, *FMR1* provides one of the larger contributions of a single gene to the dyscalculia phenotype reported so far. Similar findings have been previously reported^38^. A genetic contribution to MD has also been reported for 22q11.2 deletion syndromes^45,46^. Most importantly, our data suggest that even minor alterations of the *FMR1* gene may contribute to developmental dyscalculia.

This is the second study from our group to investigate the genetic basis of developmental dyscalculia. In the same sample, a child has already been identified with a 22q11.2(LCR4–LCR5) deletion syndrome^46^. Therefore, we found three children with a genetic condition out of 121 children with MD. None of these children had severe congenital malformations or even a funny face that would refer them for a genetic investigation. These findings are preliminary, but raise the question whether and when a genetic investigation should be considered in otherwise healthy children with MD.
